# Early Dinner Time and Caloric Restriction Lapse Contribute to the Longevity of Nonagenarians and Centenarians of the Italian Abruzzo Region: A Cross-Sectional Study

**DOI:** 10.3389/fnut.2022.863106

**Published:** 2022-03-22

**Authors:** Donato Angelino, Francesca Pietrangeli, Mauro Serafini

**Affiliations:** Faculty of Bioscience and Technology for Food, Agriculture and Environment, University of Teramo, Teramo, Italy

**Keywords:** centenarians, longevity, chrono-nutrition, caloric restriction lapse, Abruzzo, dietary habits, lifestyle

## Abstract

Recent findings showed the role of late-night eating in metabolic disorders, highlighting the importance of meal timing for health. No evidence is available on the role of meal timing for longevity. The aim of this study was to survey, in a cross-sectional study, meal timing and dietary habits of 68 nonagenarians and centenarians of the Abruzzo region, Italy. Results showed an early dinner (7:13 p.m.) and a calorie restriction lapse of 17.5 h between dinner and the following lunch. The frequency of consumption was high for cereals, vegetables, fruits, and legumes; low for meat, processed meat, and eggs; and negligible for sweets. Subjects were physically active throughout life. Our results support the importance of a daily caloric restriction lapse, hampering nocturnal postprandial stress and optimizing metabolic response, associated with high consumption of plant-based foods and physical activity for the longevity of centenarians from Abruzzo.

## Introduction

The life expectancy of human individuals is consistently increasing across the decades, almost doubling in the last two centuries (1). The World Health Organization (WHO) forecasts that the rate of 900 million people aged over 60 years will increase up to 2 billion in 2050 (2), with the number of centenarians rising from 573,000 in 2020 to 19,000,000 by 2100 (3). Aging involves a general decay of organ function, in terms of muscle and bone frailties, impairment of renal and hepatic function, as well as loss of cardiovascular and immunological functionality (4). Among lifestyle factors, the diet has been found to significantly impact the health status of individuals and, in turn, to be a key variable impacting the physiological decay of aging processes (5, 6).

Italy is the first country in Europe and the second one in the world, in terms of percentage of citizens older than 65 years of age (22.8% of the total population, following a high of 28% in Japan) and over 85 years (3.6% of the total population, following a high of 4.5% in Japan) (7). The Greek island Ikaria (8) and the Italian Sardinian “*Ogliastra*” subregion are the two locations in the Mediterranean area identified as the “Longevity Blue Zone,” for the extremely high rate of centenarians among its citizens (9). Concerning the “*Ogliastra*” region, the first hypothesis proposed to explain these demographic data concerning the genetic isolation of the Sardinian population, due to limited immigration into the island as well as to many conserved anthropological traditions (9). However, more recently, the food habits of these citizens have been taken into consideration to determine if they might have a role in longevity, both today and in the past (10, 11). In fact, their dietary habits seem to resemble the building blocks of the Mediterranean diet, known to be characterized by a high intake of plant-based foods, a moderate intake of fish and dairy products, and a low intake of meat and processed foods.

Based on the data from the Italian National Institute of Statistics (ISTAT), the region of Abruzzo, placed in center Italy, is among the top areas of Italy for the number of nonagenarians (*N* = 19,673, the 2.43% of the Italian nonagenarians) and centenarians (*N* = 485, the 2.82% of the Italian centenarians). In fact, with respect to the total population, Italian nonagenarians are the 1.37% and Italian centenarians the 0.03% (12). Particularly, the province of L’Aquila, which shows a high rate of nonagenarians and an extremely higher number of centenarians, compared with the Italian average: 0.043% vs. 0.028%, with the highest numbers occurring in the most rural, internal areas of the region (12).

The inhabitants of the Abruzzo region have typically followed a peculiarly unique dietary habit called “*sdijuno,”* representing the salty breakfast of the morning that was “breaking” the fasting of the night after an early dinner. The putative advantages of this practice are as follows: (i) negligible postprandial stress during the evening, in agreement with the circadian rhythm and lower metabolic efficiency in the night (13, 14); (ii) a period of caloric restriction from dinner to lunch. The “*sdijuno*” practice, consistently followed for decades, might have tuned the endogenous response to daily meals, optimizing both immune and metabolic responses to dietary stressors, playing a role in Abruzzo’s longevity rates. However, despite recent findings on the importance of meal timing for health (15, 16), no evidence is available on the meal timing of nonagenarians and centenarians. The aim of this study was to survey, in a cross-sectional study, mealtime, adherence to comply with “*sdijuno*” practice, as well as general dietary habits and the level of physical activity throughout life, of nonagenarians and centenarians of the Abruzzo region.

## Materials and Methods

### Study Design and Participants

A cross-sectional study was conducted in the time frame September 2019–June 2020 in the province of L’Aquila, Abruzzo Region, Italy (L’Aquila capital coordinates: 42°21′14′′N; 13°23′31′′E). The study protocol was approved by the local Ethics Committee for Human Research for the Provinces of L’Aquila and Teramo of the Local Health Authority “A.S.L. 1 – Avezzano – Sulmona – L’Aquila” (protocol no. 151856/20). The primary and secondary endpoints were those declared when the study was registered at clinicaltrials.gov (as NCT04840381). Inclusion criteria were as follows: (i) being 90 years or older at the moment of the interview; (ii) being born and living for most of one’s life in the Abruzzo region; and (iii) mentally active.

### Data Collection

Volunteers were recruited by public announcements in local newspapers, through social networks, and by personal acquaintances among the personnel of the University of Teramo. A trained nutritionist first contacted a relative of each subject to inquire about their general cognitive conditions. In positive cases, a trained nutritionist contacted the subjects – in the presence of a relative – for the interview. The interviewers fluently understood the local dialect, in order to make participants feel comfortable.

Gender, age, body weight, and height were retrieved from general questions posed to the volunteer, together with information about their health status, e.g., presence of pathologies, hypercholesterolemia, hypertriglyceridemia, hypertension, or hypotension. Other questions were related to the following: (i) the daily mealtime during the subjects’ youth and old age; (ii) the source of the food supply – own production, exchange, and local vendor; and (iii) the amount of moderate or vigorous physical activity, in terms of minutes per week, throughout life.

### Nutritional Assessment

Information on dietary intake of some foods and food groups during the subject’s youth was retrieved by means of a food frequency questionnaire (FFQ). The questions were related to the frequency of consumption, in terms of the number of times per week, of the following items: cereals, legumes, vegetables, fruit, milk and dairy products, meat, processed meat, eggs, and fish. Furthermore, questions on the use of fat during meal preparation were asked, e.g., lard, *strutto* (fat from pork), bacon, and extra-virgin olive oil. Finally, participants were asked to indicate whether they followed the *sdijuno* practice.

## Results

Demographics, anthropometric measures, and health status of the study participants are shown in Table 1. For a total of 68 volunteers, i.e., 46 women and 22 men, 46 were aged in the range of 90–99 years, while 22 were centenarians, up to 107 years of age. Subjects were all normal weight, and, except for hypertension which affected 72% of the individuals, they were characterized by an extremely low frequency of hypertriglyceridemia (4%) and hypercholesterolemia (19%).

**TABLE 1:** Demographic, anthropometric, and health status of the study participants.

Total (*N*)	68
Female	46
Male	22
**Age (*N*)**	
Nonagenarian (*90–99 y*)	46
Centenarian (*100–107 y*)	22
BMI (*kg/m^2^*)	24.8 ± 0.4
**Main pathologies (*%*)**	
Diabetes	13
Hypercholesterolemia	19
Hypertriglyceridemia	4
Hypertension	49
Hypotension	3

*BMI, body mass index, expressed as mean ± SEM.*

**FIGURE 1:**
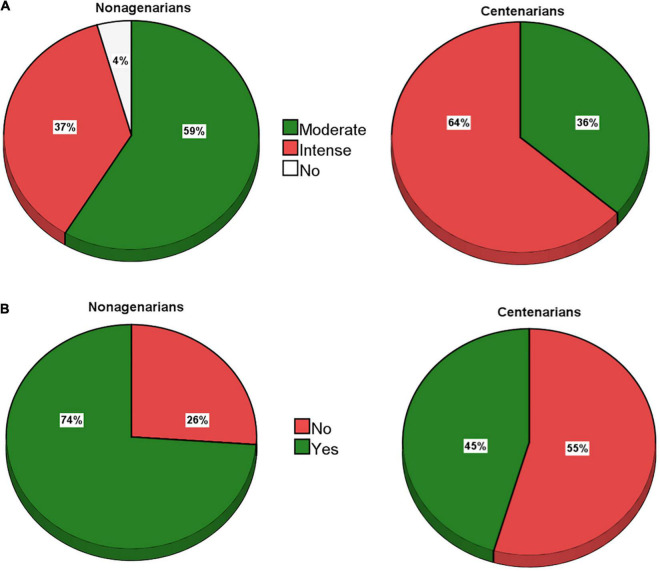
Physical activity status of the study participants in their youth **(A)** and elderly **(B)** age.

Almost all the subjects had been physically active throughout life, and most of them were still active at the time of the interview, refer to Figure 1.

Notably, 59 and 36% of the nonagenarians and centenarians, respectively, stated that they had been moderately physically active in their youth, i.e., walking, biking, and horse riding (data not shown). Again, 37 and 64% of the nonagenarians and centenarians, respectively, reported intense physical activity earlier in life, due to the long hours spent on their work activity, i.e., work in the fields of animal husbandry in mountainous areas (data not shown). In fact, only 3% of the nonagenarians and none of the centenarians reported less or no significant physical activity earlier in life (Figure 1A). Concerning the present level of physical activity, reported in Figure 1B, 74% of the nonagenarians and 45% of the centenarians still practiced moderate physical activity, mainly walking or taking care of the garden or homeworking (data not shown). The remaining 26% and 55% of nonagenarians and centenarians, respectively, did not report their physical activity, mainly due to their deteriorating physical conditions or due to permanent wheelchair use (data not shown).

**FIGURE 2:**
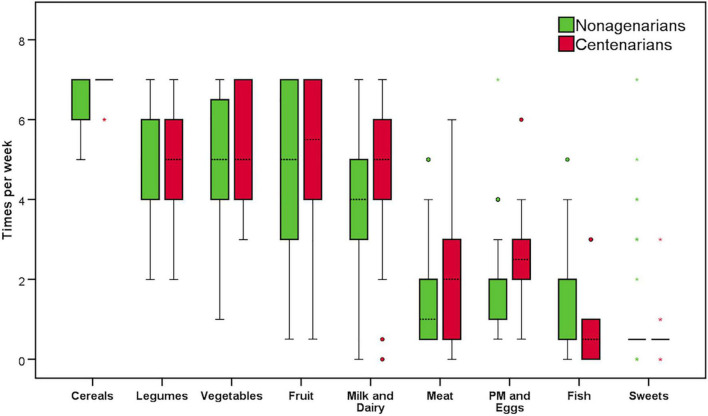
Frequency of consumption of food groups in the nonagenarian and centenarian populations. PM, processed meat. Circles and asterisks represents outlier and extreme outlier values, respectively.

Data retrieved from the subjects’ dietary habits in their young age, in terms of frequency and serving size, split for their decade of age, are summarized in Figure 2. Results were very similar among nonagenarians and centenarians, with a median intake of five or more servings of each of several plant-based foods per week, such as cereals, legumes, vegetables, and fruit, with the lowest consumptions accounting for 1–2 servings per week. Among the animal-based products, milk and dairy products were consumed, in moderation, four and five times per week by nonagenarians and centenarians, respectively. Concerning meat and fish products, consumption was only one to two servings per week, while processed meats and eggs had a median consumption of two to three servings per week in the whole population. Lastly, consumption of sweets was negligible, with a median intake of one serving per week and mostly on Sundays or festive days (data not shown).

**FIGURE 3:**
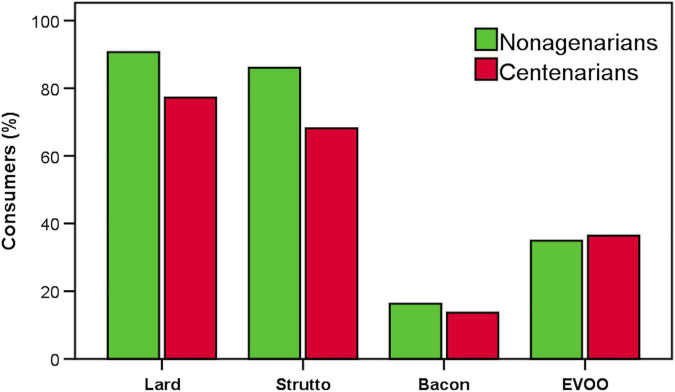
Percentage of consumer use of cooking fat during meal preparation. EVOO, extra-virgin olive oil.

Subjects were also asked to report the main seasonings used during the preparation of meals. Figure 3 shows that a higher number of nonagenarians and centenarians mainly used animal-derived fats compared with extra-virgin olive oil, used by 34 and 36% of nonagenarians and centenarians, respectively. In fact, more than 84% of the nonagenarians and 68% of the centenarians, respectively, used lard and *strutto* for the preparation of recipes, while bacon (also including the Italian *pancetta*) was used by 16 and 14% of nonagenarians and centenarians, respectively.

The survey of the condiments also included questions about the main spices used by participants during food preparation. On the whole, both nonagenarians and centenarians most often used rosemary, garlic, onion, chili pepper, parsley, and basil (data not shown).

**FIGURE 4:**
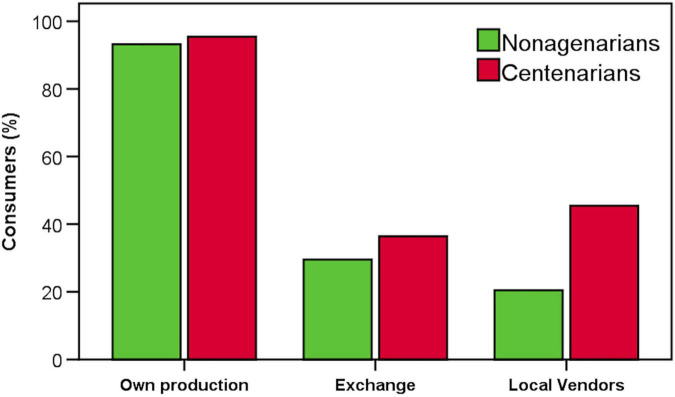
Percentage of subjects consuming foods produced by themselves, exchanged with other locals, or bought from local vendors.

Since the subjects resided in rural and relatively inaccessible areas, we also investigated the way of the food supply. Figure 4 shows that almost 95% of the nonagenarians and centenarians made their own meals from scratch, while 30 and 36% of the nonagenarians and centenarians, respectively, used foods exchanged with other people in the neighborhood or the surroundings. Finally, 20 and 46% of the nonagenarians and centenarians, respectively, bought foods only from local vendors.

**FIGURE 5:**
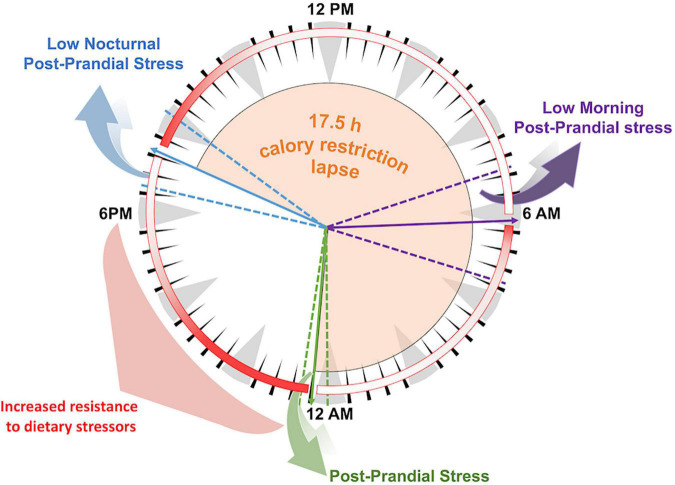
Graphical representation of the day’s mealtimes and of the fasting/calorie restriction lapse for nonagenarians and centenarians from Abruzzo. The arrows within the clockface indicate the median time of meal consumption: purple: breakfast; green: lunch; and blue: dinner. Dotted lines represent the earliest and latest time of each meal consumption. Light-orange areas represent an average time of restricted calorie intake. Red shaded circle areas represent hypothesized postprandial stress periods after each meal, the bolder the red, the greater the postprandial stress.

A very high percentage of the subjects – 90% of the total – stated that they had adhered to the *sdijuno* practice (data not shown). Regarding meal timing, dinner, consisting mainly of vegetable soups, polenta, vegetables, eggs, or cheese, was consumed on average at 7.13 p.m. The breakfast meal was on average at 6:18 a.m. and included dishes leftover from dinner, milk and bread, or a slice of bread with ham. The main meal of the day was lunch, composed of meat, pasta or polenta, and beans, and was on average at 12.38 p.m., highlighting a caloric restriction period of approximately 17.5 h between dinner and the following lunch (Figure 5).

## Discussion

In this study, we provided novel studies about the importance of meal timing and of a caloric restriction period of approximately 17–18 h, together with physical activity and a dietary pattern based on high consumption of plant-based foods, moderate consumption of the animal product, and negligible consumption of sweets, for the longevity of Abruzzo’s centenarians.

Epidemiological studies have pointed out to a late-night dinner being associated with an increased incidence of cardiovascular diseases, i.e., stroke, heart failure, etc. (17). These findings have been confirmed in two intervention studies, where Japanese volunteers, affected by type-2 diabetes, showed lower postprandial glycemia and insulinemia when they ate their main meal at 6 p.m., compared with 9 p.m. (18, 19). The authors discussed these important findings, emphasizing that just a 3-h shift of the mealtime resulted in a significant health benefit was quite remarkable. This implicates the importance of the circadian rhythm, as “*the diurnal variation in insulin resistance is higher at night than in the daytime*,” and that the long fasting period from lunch to late dinner disrupted this (19). These latter aspects induce an increase of the plasma-free fatty acid values and a decrease of the blood insulin concentrations, leading to a higher postprandial glycemia and metabolic stress (19). Thus, the metabolic impact due to non-optimal eating behaviors likely increases the risk for cardiovascular events, playing a role in life expectancy.

As drawn in the biological clock in Figure 5, “*sdijuno*” implies a ∼17.5 h period of low caloric intake, which might be considered a “time-restricted feeding” regimen, under the frame of the intermittent fasting and calorie restriction (20, 21). If we analyze the proposed metabolic postprandial stress periods after each of the three meals of the day (Figure 5, detailed in red), an early dinner causes minimal nocturnal stress, as does the low-calorie intake at breakfast (200–300 kcal). The daily caloric restriction lapse might allow both metabolism and the immune system to efficiently minimize the stress induced by the main meal of the day. Despite very few available human intervention studies, recent findings show that time-restricted feeding has a positive role in the control of insulin sensitivity and β-cell responsiveness, through modulation of the gut microbiota profile and metabolic products and through the alignment of activity of the microbiome with the circadian rhythm (22), confirming previous suggestions of the importance of biological rhythms in health. This metabolic virtual cycle, recurring each day in the life of the centenarians, may have positively affected both glycemic control and lipid metabolism, playing an important role in their longevity.

We showed that subjects had an almost daily consumption of plant-based foods, above all cereals, legumes, fruits, and vegetables, and moderate weekly consumption of milk and dairy in agreement with the consumption pattern of the Mediterranean diet (23) with the exception of the frequent use of animal fats instead of olive oil. Our findings are in agreement with the results of a recent study conducted in the Italian Blue Zone of Sardinia, showing that individuals in the range of 90–101 years old had a higher intake of lard over olive oil (3.8 times/week vs. 2.51 times/week, respectively) (11). As L’Aquila rural area is particularly mountainous, olive trees are not a local culture, and the inhabitants had their own production of foods from the ground and with animal breeding, and it is reliable to think that the consumption of animal fats is higher than the olive ones. In fact, the 20%–40% of the subjects who chose to use vendors for their food supply were also those consuming olive oil. Last, but not least, being olive oil imported from surroundings, it could have resulted more expensive than pork-based ones, letting the product not affordable.

Median intakes of other food groups consumed by Abruzzo elderlies are almost overlapping with the ones found for Sardinian Blue Zone inhabitants (11). Among these, cereals and cereal-based foods are confirmed to be almost daily consumed by all the Sardinians elderlies, as in the Abruzzo ones; regarding other plant-based products, legumes and vegetables are also consumed on average ∼4 times per week. The only difference has been pointed out for fruits, which in the Abruzzo elderlies was found to be consumed with a double frequency compared with Sardinian ones. Also, animal-based food categories mostly agreed in terms of frequency of consumption of the following: milk and dairy products ∼4 times per week, meat between 1 and 2 times per week, and fish less than 2 times per week (11). In addition, we found an abundant and frequent use of herbs and spices during meal preparation – data missing in Sardinian Blue Zone related papers – which may have had a role in the counteraction of inflammatory-related disorders due to their content in bioactive compounds (24).

During the interview, elderlies shared their main recipes prepared and consumed during the three main meals of the day. Among these, breakfast was usually what in Italy is known as “*salted breakfast*,” with the only sweet-tasting item being milk – although it was not consumed daily– and with most of the dishes done with the dinner leftovers, i.e., *polenta*, potatoes, stuffed leafy vegetables and bread spread with animal fats, cheese, and eggs. The lunch, which was the main meal of the day consumed during the working activities, consisted of home-made egg-based recipes or whole wheat pasta with vegetables or *polenta* and soups made with local cultivars of lentils, beans, chickpeas (among which the local spread *cicerchia*, alias *Lathyrus sativus)* or meat, such as lamb or beef. Dinner was mainly based on vegetables or soups or *polenta* and some other animal-based products, i.e., own pecorino cheese, sheep ricotta. Fish was mainly present with local species from the surrounding river or (salt) cod, consumed with lower frequency than meat. Last, but not least, the sugar consumption was close to zero, with sweets or cakes eaten only on special occasions.

A further aspect related to their longevity is the high level of physical activity for most of the nonagenarians and centenarians. This is mainly linked to their work on the land for most of the day, as also found in a Polish survey about the dietary and lifestyle habits of Warsaw centenarians (25). More than 50% of the nonagenarians and the centenarians remained physically active, even at the time of the interview, walking around their community and even still doing some light work on the land, maybe supporting the advantages of regular physical activity throughout life for healthy longevity (26, 27).

It is worth highlighting the limitations of the present survey. First, being a small study and limited to a single province of the Abruzzo region, data retrieved from the few interviewed individuals may not be applicable for all the Abruzzo centenarians and nonagenarians as well as for individuals out of region and country. Then, our study is based on a retrospective survey of nonagenarians and centenarians who gave information on their food habits, dating back sometimes only 60 years, which barely covered their youth. So, there is a bias relative to their physiological loss of clarity in their memories and another in “summarizing” the food habits in a unique answer.

Concerning food habits, the main limitation to this study is the physiological loss of precision in the memories of dietary and lifestyle habits. Moreover, FFQ does not allow any quantitative assessment, and, as previously found (11), this may result in only a rough grouping of foods consumed by the subjects in their youth, yet their memories are supported by the availability of local ingredients and food preparation methods passed down within families.

## Conclusion

Our findings support the importance of a daily caloric restriction lapse, associated with high consumption of plant-based foods and physical activity for the longevity of centenarians from Abruzzo. Despite more studies needed, our results support the importance of considering meal timing as a feature involved in longevity processes.

## Data Availability Statement

The raw data supporting the conclusions of this article will be made available by the authors, without undue reservation.

## Ethics Statement

The studies involving human participants were reviewed and approved by Ethics Committee for Human Research for the Provinces of L’Aquila and Teramo of the Local Health Authority “A.S.L. 1 – Avezzano – Sulmona – L’Aquila.” The patients/participants provided their written informed consent to participate in this study.

## Author Contributions

DA was involved in the data analyses, interpretation of the results, and drafting of the manuscript. FP was involved in the protocol design and data collection. MS conceived the study and involved in the protocol design, interpretation of the results, drafting of the manuscript, and had primary responsibility for the final content. All authors contributed to the article and approved the submitted version.

## Conflict of Interest

The authors declare that the research was conducted in the absence of any commercial or financial relationships that could be construed as a potential conflict of interest.

## Publisher’s Note

All claims expressed in this article are solely those of the authors and do not necessarily represent those of their affiliated organizations, or those of the publisher, the editors and the reviewers. Any product that may be evaluated in this article, or claim that may be made by its manufacturer, is not guaranteed or endorsed by the publisher.
